# Mammarenaviruses depend on endogenous fatty acid synthesis in cell culture systems

**DOI:** 10.1016/j.jlr.2026.101038

**Published:** 2026-04-14

**Authors:** Joseph Thomas Noble, Maryam Siddique, Kingsley Bimpeh, Nathaniel Jackson, Amaani Ibrahim, Thomas Lucien McDaniel, Nathan W. Davis, Caitlin Bone Wade, Luis Martínez-Sobrido, Kelly Marie Hines, Melinda Ann Brindley

**Affiliations:** 1 Department of Infectious Diseases, University of Georgia, Athens, GA, USA; 2 Department of Chemistry, University of Georgia, Athens, GA, USA; 3 Texas Biomedical Research Institute, TX, USA; 4 Department of Population Health, University of Georgia, Athens, GA, USA

**Keywords:** Lassa, lipids, mammarenavirus, fatty acids, FASN

## Abstract

Lassa virus (LASV) and lymphocytic choriomeningitis virus (LCMV) are Old World mammarenaviruses that, like all viruses, rely on host-derived biological molecules to complete their replication cycle. Identifying host factors essential for mammarenavirus replication may reveal novel targets for antiviral intervention. To this end, we found that replication of recombinant tri-segmented r3LCMV and r3LCMV LASV chimera (r3LCMV-LASV) was sensitive to reductions in both exogenously supplied and endogenously synthesized lipids. Lipidomic analysis on mock-infected and virus-infected VeroS cells revealed infection with r3LCMV-LASV increased the abundance of triacylglycerols (TG), phosphatidylcholine (PC), and phosphatidylglycerol (PG), while decreasing levels of ceramides, phosphatidylethanolamine (PE), and phosphatidylserine (PS). Although TG levels rose during infection, pharmacologic inhibition of TG synthesis did not impair viral replication. In contrast, inhibition of fatty acid synthase (FASN), a key enzyme upstream of TG synthesis, significantly reduced r3LCMV-LASV and r3LCMV spread. FASN inhibition suppressed both viral genome replication and viral budding. The addition of oleic acid, but not palmitic acid (the principal product of FASN), rescued the inhibitory effect of FASN blockade. Reducing FASN using shRNA conferred similar effects. Dependence on FASN activity was observed across multiple New and Old World mammarenaviruses and in multiple different cell lines, further reinforcing the importance of fatty acid synthase for productive mammarenaviral infection.

As obligate intracellular parasites, successful virus reproduction relies on multiple elements of cellular metabolism, including nucleotide, protein, and lipid synthesis pathways (1). Genome replication of positive-sense RNA viruses occurs on membranous structures, and studies show replication induces large changes in the cellular lipidome initiated by the formation of these replication structures (2, 3, 4). While fewer studies have examined how negative-sense RNA viruses alter the cellular lipidome, cells infected with influenza, Uukuniemi virus, and vesicular stomatitis virus (VSV) display altered lipidomes, suggesting infection impacts lipid metabolism even when dramatic changes to the cellular membrane architecture do not occur (5, 6, 7, 8, 9). How the altered lipidome influences the successful replication of the virus and if certain lipid species are required for viral reproduction largely remains unknown. Lipid metabolic pathways intersect with many other cellular pathways and therefore alterations in lipid levels may not only influence obvious lipid modifications such as changes to membrane fluidity, but can also alter cellular ATP pools, protein localization, post-translational modifications, cell division, histone acetylation, and gene expression (10, 11).

Lymphocytic choriomeningitis virus (LCMV) and Lassa virus (LASV) are enveloped viruses in the *Arenaviridae* family. Human cases of LCMV and LASV arise from zoonoses, as the common “house” mouse, *Mus musculus,* is the primary host for LCMV, and the “multimammate” rat, *Mastomys natalensis*, is the reservoir for LASV. Infected host rodents excrete viruses within their urine and fecal waste (12). Humans can become infected from exposure to aerosolization or direct contact with virus laden waste. LASV and LCMV are the causative agents of Lassa fever (LF) and lymphocytic choriomeningitis (LCM), respectively. Serological studies for LCMV and LASV suggest most infections are mild or asymptomatic, however severe cases can arise especially in immunocompromised individuals and pregnant women (13, 14). When symptomatic, LCMV infection generally manifests as an initial febrile illness that can progress to aseptic meningitis or encephalitis. In contrast, LASV infection is often presented with nonspecific febrile symptoms that, in severe cases, may advance hemorrhagic manifestations. Following recovery, as many as 30% of LF cases are accompanied by idiopathic deafness (15). These complications from illness and a lack of FDA-approved vaccines and specific antiviral drugs led to classifying LASV as a biosafety level (BSL) 4 pathogen and select agent.

Both LCMV and LASV are Old World (OW) mammarenaviruses that contain bi-segmented, negative-sense RNA genomes that encode for 4 proteins using an ambisense coding strategy. The large (L) genome segment encodes the RNA-dependent RNA polymerase (RdRp) and the matrix protein (Z). The small (S) genome segment encodes the nucleoprotein (NP) and the glycoprotein complex (GPC). The enveloped viral particles bind to alpha-dystroglycan, or other attachment factors, and are macropinocytosed into the cell (16, 17, 18, 19). GPC mediates the fusion of the viral and cellular lipids upon exposure to low-pH in the lysosome (20, 21). Viral genome replication occurs in the cytoplasm, requiring both viral RdRp and NP (22). Eventually, Z protein mediates newly formed viral particle budding from the plasma membrane (23).

Both OW and New World (NW) mammarenavirus particle production is dependent upon protein acylation. The Z and GPC proteins of LCMV and LASV are myristoylated, meaning a myristate, a 14-carbon saturated fatty acid, is covalently attached to the amine-groups of a glycine residue at the N-terminus by N-myristoyltransferases (NMT) (24). Myristoylation, along with neighboring hydrophobic residues, helps localize proteins to membranes (25). Mutations of the glycine in the second position to alanine (G2A) decrease mammarenavirus budding, and similarly, blocking myristylation by inhibiting NMT also disrupts arenaviral budding (24, 26). Myristoylation of the stable signal peptide (SSP) of LCMV and Junín (JUNV) GPC, a NW mammarenavirus, is not needed for efficient budding, but is critical for fusion (27, 28, 29). In addition to the fatty acid modifications on two of the four viral proteins, previous studies have identified other key roles for lipids during arenaviral entry. Lysosome-associated membrane protein 1 (LAMP1) facilitates LASV GPC-mediated conformation changes in a cholesterol-dependent manner (30). In addition, LASV fusion pore expansion is promoted by cellular bis(monoacylglycero)phosphate (BMP), an anionic lipid enriched in late endosomes (31).

Here, we expand the current understanding of viral lipidomics by demonstrating how mammarenaviruses both depend on and significantly remodel the host cellular lipidome. Despite the accumulation of triacylglycerols (TGs) during infection, viral replication was not dependent on TG synthesis. Instead, replication required metabolic processes upstream of TG formation, specifically at the level of fatty acid synthesis. Inhibition of fatty acid synthase markedly reduced the production of infectious particles, viral RNA synthesis, and budding efficiency. Importantly, this inhibition was reversed by supplementation with oleic acid, suggesting that mammarenavirus replication and budding may rely on complex lipids containing unsaturated acyl chains.

## Materials and Methods

### Cell lines

Vero cells (ATCC CCL-81), VeroS (vervet green monkey kidney cells producing human SLAM) cells (32), and BSR-T7 (BHK-21 cells producing the T7 polymerase) (33) were maintained in Dulbecco’s Modified Eagle Medium (DMEM) with 5% FBS. Human HEK293T (ATCC CRL-11268) cells, mouse hepatocarcinoma-derived Hepa1-6 cells (ATCC CRL-1830), a kind gift from Dr Sam Kurup from the University of Georgia, human liver carcinoma Huh7 cells, a kind gift from Wendy Maury from the University of Iowa, and human lung adenocarcinoma A549 cells (ATCC CCL-185) were maintained in DMEM with 10% FBS. All cell lines were maintained at 37°C and 5% CO_2_ and were periodically tested for mycoplasma using the PlasmoTest™—Mycoplasma Detection Kit (InvivoGen, cat. rep-pt1) and remained negative throughout all experiments. All human and mouse cell lines used within this study have been authenticated through STR profiling through LabCorp Cell Line Authentication Services, followed by STR Analysis with CLASTR 1.5.0.

### Viruses

The r3LCMV and r3LCMV-LASV were generated using previously described reverse genetics systems (34). Briefly the S gene segment was duplicated. In one copy the GPC was replaced with Gaussia-luciferase (GLuc), and on the other copy, NP was replaced with GFP. Infectious virus requires both S segments within the virion. The r3LCMV-LASV was produced by replacing the LCMV GPC with the LASV GPC. The r3LASV, Josiah strain and the r3JUNV, Romero strain were previously described (26, 35) and were generated using similar reverse genetics (34). Viral stocks were amplified in BSR-T7 cells. Tacaribe virus (TACV) (ATCC VR-1272), ML29, a kind gift from Dr Peter Pushko at Medigen, and Junin candid1, a kind gift from Dr Slobodan Paessler from the University of Texas Medical Branch, were amplified in VeroS cells. Virus titer was determined by calculating the 50% tissue culture infective dose (TCID50) units per mL through end-point dilution using the Spearman-Karber method on VeroS cells.

### Lipid-altering compounds

Compounds were purchased from Cayman Chemical and resuspended in anhydrous DMSO at the concentrations indicated, unless otherwise indicated. Cetaben (cat#10007171, 50 mM), Afatinib (cat#11492, 25 mM), Rosiglitazone (cat#71740, 100 mM), GW3965 (cat#10054, 30 mM), A922500 (cat#10012708, 50 mM), T863 (cat#25807, 50 mM), NG497 (cat#36886, 18 mM), Atglistatin (cat#15284, 75 mM), CAY10499 (cat#10007875, 50 mM), CTPI-2 (cat#36933, 10 mM), ND646 (cat#34764, 50 mM), TOFA (cat#10005263, 30 mM), PF-05175157 (cat#21778, 50 mM), TVB-2640 (cat#35703, 50 mM), GSK2194069 (cat#20022, 33 mM), IMP-1088 (cat#25366, 10 mM in methyl acetate), and CVT12012 (cat#27156, 50 mM).

### Drug assays

Vero cells were plated at 50,000 cells/well in 96-well plates. The following day cells were treated with compounds at the indicated concentrations or similar concentrations of DMSO for control in triplicate. Immediately after treatment, cells were infected with r3LCMV-LASV or r3LCMV (MOI 0.01). Infected cells were incubated for 48 h, then luciferase was quantified using the Gaussia Luciferase Glow Assay (Thermo Scientific), or drug cytotoxicity was evaluated in duplicate plates lacking virus using CellTiter Glo (Promega). The average results from the triplicate-treated cells were compared to the average results from DMSO controls. The experiment was repeated at least three independent times.

### Lipid extraction

VeroS cells plated in 10 cm dishes were infected with r3LCMV-LASV (MOI 0.05) or mock-infected for 1 h, when the inoculum was removed, and fresh media were replaced. Cells were prepared for lipid extraction at 72 hours following infection, when 95% of the cells were GFP positive. Cells were washed in ice-cold PBS two times and quickly scraped in 1 ml of PBS. The suspension was transferred to a glass tube and sonicated in an ice bath for 30 min. Lipid extraction was performed using a modified version of the Bligh and Dyer extraction method (36, 37, 38). Cold chloroform/methanol (2:1 v/v) was added to the samples and vortexed intermittently for 5 min on ice. An additional 1:1 volume of chloroform and water was added to the suspension in a glass tube and vortexed for 1 min. The resulting mixture was centrifuged (10 min, 2000 *g*, 4°C). The organic layer was transferred to a new glass tube and dried in a speed-vac concentrator. The dried lipid extracts were subsequently reconstituted with 1:1 chloroform/methanol (v/v) and stored at −80°C.

### HILIC-IM-MS

Lipid extracts were analyzed using an online Hydrophilic Interaction Liquid Chromatography (HILIC) coupled with a Waters Synapt XS Travelling Wave Ion-Mobility Mass Spectrometry (TWIMS-MS). Chromatographic separation was performed at 40°C with Waters Cortecs Ultra Performance Liquid Chromatography (UPLC) HILIC column (2.1 × 100 mm, 1.6 μm) connected to Waters Acquity FTN I-Class Plus UPLC. Mobile phase A (MPA) consisted of 50% acetonitrile and 50% water with 10 mM ammonium acetate, and mobile phase B (MPB) consisted of 95% acetonitrile and 5% water with 10 mM ammonium acetate. Using a fixed flow rate of 0.5 ml/min, lipids were separated using the following gradient method:1–2 min, 100% MPB; 3–4 min, 60% MPB; 5–7 min, 100% MPB. Lipid extracts were dried in a vacuum concentrator (Savant Thermofisher) and prepped at 15X dilution with MPB. Quality control (QC) samples were prepared by pooling equal volumes from each sample. All prepped samples were stored in an autosampler maintained at 6°C to maintain sample integrity over the LC-MS run. All experiments were performed with an injection volume of 5 μl.

Mass spectra were acquired in positive and negative ion mode with the following electrospray ionization (ESI) source conditions: capillary voltage, (+/−) 2.0 kV; sampling cone voltage, 40 V; source offset, 4 V, source temperature, 135°C; desolvation temperature, 500°C; desolvation gas flow rate, 1000 L/h; cone gas flow rate, 50 L/h. TWIM separations were performed in nitrogen with a gas flow of 90 ml/min, wave velocity of 550 m/s, and wave height of 40 V. TWIM separations were performed in nitrogen with a gas flow of 90 ml/min, wave velocity of 550 m/s, and wave height of 40 V. Mass calibrations were performed with sodium formate over the range of 50–1,200 m/z. Continuum data were collected with a 0.5 s scan time over the range of 50–1,200*m/z* with the time-of-flight (TOF) mass analyzer operating in resolution mode (resolution of ∼30,000). Leucine-enkephalin was continuously infused over the entire acquisition time for lock mass correction. Ion mobility-organized data-independent acquisition (MS^E^) was performed in the post-ion mobility transfer region of the instrument with a 35–50 eV collision energy ramp.

### Mass spec data analysis and processing

Data processing was performed in Progenesis QI software (v3.0, Waters/Nonlinear Dynamics). Samples were grouped into their respective categories after peak picking and lock-mass correction with leucine enkephalin lock-mass signal. Principal Component Analysis (PCA) was performed with EZinfo (v3.0, Umetrics) on the data set filtered by an ANOVA *P*-value indicated in the PCA plots. Orthogonal Projection to Latent Structures Discriminant Analysis (OPLS-DA) with S-Plot was carried out on the filtered data to identify lipids that were statistically different from the investigated groups. Lipid identifications were carried out using retention times and accurate mass (<10 ppm) compared with an in-house database developed by LipidPioneer. Mass spectrometry data are available at MassIVE, under Accession MSV000100137.

### Lipids

To account for poor solubility and bioavailability as free fatty acids, myristic (C14:0, cat#13351), palmitic (C16:0, cat#10006627), oleic acid (C18:1, cat#90260), and linoleic (C18:2, cat#90150) (purchased from Cayman Chemical) were dissolved in 100% ethanol and were conjugated to immunoassay-grade BSA (Thermofisher cat#J65731.22), to a fatty acid-to-BSA ratio of 2 mM–0.4 mM (1X), as prepared in (39). Ethanol was applied at 0.17% v/v (1/10x), myristic acid, oleic acid, and linoleic acid at 200 μM (1/10X), and palmitic acid was utilized at 100 μM (1/20X) to account for qualitatively-assessed cytotoxicity, which has been reported before for palmitic acid (40).

### HCS LipidTox red staining

VeroS cells were plated on glass coverslips and allowed to adhere overnight. Cells were either treated with oleic acid or oleic acid + A922500 or infected with r3LCMV or r3LCMV-LASV (MOI 0.01). Two days following infection cells were fixed in ice-cold formalin (3.7%) for 20 min, washed in cold PBS twice, and stained in 500 μl of HCS LipidTox Red Neutral Lipid Stain (1/1000X) for 30 min (ThermoFisher cat# H34476), followed by nuclear staining with 1 μg/ml Hoechst 33,342 for 15 min. Cells were washed in PBS, mounted onto slides with ProLong Diamond Antifade Mountant (ThermoFisher), and imaged with a Nikon A1 confocal (60X Oil Immersion).

### Viral spread assay

VeroS cells were plated at 2.5 × 10^5^cells/ml using DMEM+ 5% FBS. At 24 h post seeding, cells were infected with r3LCMV-LASV (0.001 MOI). One hour after infection, the infection inoculum was removed and DMEM-5% FBS with 33 μM GSK2194069 was added. Cells were collected at the indicated time points and fixed in 3.7% formalin. GFP cells were enumerated by flow cytometry to determine the number of positive live virus cells within the population of 10,000 live, single cell events.

### Entry assays

VeroS were plated at 5 × 10^5^cells/ml in 24 wells plates. Twelve hours following seeding, cells were treated with the compounds at the indicated concentrations in DMEM + 5% FBS. After 18 h of treatment, cells were infected with r3LCMV-LASV or r3LCMV for 1.5 h before the infection inoculum and compound was removed, cells washed with DMEM-free, and media was replaced with DMEM + 5% + 10 mM NH_4_Cl. Cells were harvested with trypsin 12 hpi, fixed in 3.7% formalin, and analyzed using flow cytometry.

### Single-step luciferase assay

VeroS cells were plated at 2.5 × 10^5^cells/ml, then infected r3LCMV-LASV or r3LCMV (MOI 0.5) for 1 h. Virus was removed, cells were washed with PBS, and media was replaced with DMEM+5% FBS + 10 mM NH_4_Cl with compounds or DMSO at the indicated concentration. Sixteen hpi, supernatants were collected, centrifuged to remove cell debris, and assayed for Gaussia luciferase using the Gaussia Luciferase Glow Assay (Thermo Scientific).

### Western Blot-based budding assay

293T cells were plated at 2.5 × 10^5^cells/ml and the following day were transfected with the indicated pcDNAintron-Z-FLAG expression plasmid using JetPrime transfection reagent. The transfection mixture was removed after 4 h and replaced with complete media supplemented with indicated compounds or DMSO for control. Twenty-four hours following treatment, supernatants were collected and cells were lysed in M2 Lysis Buffer (150 mM NaCl, 1 mM EDTA, 50 mM Tris-HCL (pH 7.4), 1%v/v Triton X-100). Total cell lysates (TL) were cleared via centrifuge (4°C, 17,000 *g*, 25 min), and soluble material was denatured in 4 M urea with SDS (56°C, 30 min). Supernatants were cleared via centrifugation (4°C, 17,000 *g*, 25 min), protein was precipitated by adding trichloroacetic acid (TCA, 10% final volume), washed in pure acetone, and finally the pellet was resuspended in 8 M urea/10%w/v SDS and denatured (56°C, 30 min). Proteins were separated on NuPAGE Bis-Tris (4%–12%) gels, transferred onto PVDF, blocked in 10% milk, and probed with anti-FLAG (1:5000, M2, Sigma,) and anti-GAPDH (1:2000, Santa Cruz Biotechnology cat#0411). Secondary (1:10,000, mouse light-chain-specific HRP) were visualized with SuperSignal West Dura ED or Femto MS substrate on a BioRad ChemiDoc XRS+. Quantification by band volume was assessed by ImageLab, with relative budding = supernatant/Tl/GAPDH.

### Confocal with immunostaining

VeroS cells were transfected via JetOptimus with pcDNA-LASV-Z-FLAG, pcDNA-LASV-Z-FLAG-G2Aor pcDNA-LCMV-Z-HA-YFP for 5 h before transfection inoculum was removed and cells were treated with DMSO (0.1%v/v), TVB-2640 (33 μM), or IMP-1088 (1 μM) for 24 h. Cells were fixed in ice-cold formalin (3.7%v/v) for 20 min, quenched in 50 mM glycine in PBS for 30 min, and with the exception of pcDNA-LCMV-Z-HA-YFP, permeabilized in permeabilization buffer (0.1%v/v Triton X-100, 2%w/v BSA, 0.02%w/v sodium azide, PBS) at RT for 20 min. After washing in PBS, cells were blocked in blocking media (10%w/v BSA, 0.02%w/v sodium azide, PBS) for 1 h, followed by incubation in FLAG staining buffer (1:1,000X anti-FLAG-AF647 (Invitrogen cat#701629RP647), 2%w/v BSA, 0.02%w/v sodium azide, PBS) overnight. All groups were nuclear stained with 1 μg/ml Hoechst 33,342 in PBS for 15 min. Cells were washed in PBS, mounted onto slides with ProLong Diamond Antifade Mountant (ThermoFisher), and imaged with a Nikon A1 confocal at 60X, with oil immersion lenses and excitation at 405 nm and 600 nm lasers with appropriate detection filter sets. For measurement of intranuclear LASV Z-FLAG or LCMV-Z-HA-YFP, binary layers were produced by thresholding AF647/YFP and Hoechst 33,342 intensities, followed by creation of a binary layer using the intersection and automated measurements functions in NIS-Elements.

### Lipid order with NR12S and Laurdan

VeroS cells were treated with DMSO (0.1%v/v) or TVB-2640 (33 μM), with 1/20X ethanol-BSA or oleic acid-BSA, to determine whether lipid order is altered with FASN inhibition and restored by fatty acid supplementation. Methyl-β-cyclodextran (MβCD, 5 mM) was employed as a control for decreased lipid order. Treated cells were harvested at 48 h post treatment by trypsinization; 200 nM NR12S, (Cytoskeleton Inc., cat#MG08) in DMEM was applied to suspended cells and incubated at RT with rotation for 30 min. Live cell plasma membrane lipid order was assessed by flow cytometry through excitation at 488 nm, followed by detection of Red (615/20) and Green (586/20) filter sets. The reported Ratio (Green/Red) = green MFI/red MFI. Laurdan (50 μM) staining of cells was performed in a similar manner to NR12S, and flow cytometric analysis was performed on a Novocyte Penteon with 405 nM excitation and detection with a Blue (445/45) and Green (525/45) filter set. Ratiometric analysis was performed by dividing the Green MFI by the Blue MFI of each sample.

### Knockdown cell line generation

Knockdown cell lines were produced through transduction of A549 and Huh7 cell lines with lentiviral particles carrying either scramble or FASN short-hairpin RNA loci. To generate the lentiviral particles, 293T cells were transfected with TransIT-VirusGEN (Mirus Bio) reagent complexed with gag/pol (pMDLg/pRRE, Addgene #12251), VSV-G (41), tat/rev (pcDNAintron-HIV-Tat-IRES-Rev), and the transfer plasmid pLKO.1-puro-humanU6-shRNA-FASN (Addgene #82327), for shFASN cell lines, or pGIPZ Non-Silencing Control DNA (Dharmacon), for scrambled cell lines. Three days post-transfection, supernatants were harvested, cleared through centrifugation at 6,000 *g* for 5 min, and lentiviral stocks were applied to A549 and Huh7 cells. Transduced cells were selected in DMEM 10% FBS with 3 μg/ml puromycin. Selection of scrambled was confirmed by flow cytometry, as pGIPZ transfer plasmid encodes TurboGFP. Knockdown of FASN was confirmed by RT-qPCR by SYBRGreen (Thermofisher) with the following primer set: Forward (5′-TCGTGTTGACTTCTCGCTCC-3′) and Reverse (5′-CCATCTCTCAAGACCACGGC-3′). As an endogenous control, we measured ATP8B2 transcripts using the following primer set: forward (5′- gggagagaggcctgaacctg -3′) and reverse (5′ -gaagtccaggatggccagcag-3′). Percentage of FASN transcripts in shRNA cells relative to scramble cell lines was calculated using the 2^(-ΔΔC^_t_^)^ method (42).

### Dose–response inhibition of r3LASV and r3JUNV

A549 cells (96-well plate format, technical quadruplicates) were infected with r3LASV (MOI 0.003) or r3JUNV (MOI 0.006). After 1 h of viral adsorption, indicated concentrations of inhibitors were added. At 72 h (LASV) or 96 h (JUNV) post-infection, cell culture supernatants were collected, and Gluc activity was quantified according to the manufacturer protocol using Pierce™ Gaussia Luciferase Glow Assay Kit (16,160, Thermo Fisher Scientific) and GloMax plate reader (Promega). Data were normalized to mock-treated, infected cells. Cells were then fixed in 10% formalin and imaged for GFP and DAPI expression using an EVOS M5000 imaging system (Thermo Fisher Scientific). IC50 calculated with four-parameter logistic regression.

### Statistics

Statistical analyses were performed using Prism (GraphPad). Normality checks for Gaussian residuals were evaluated using the Shapiro–Wilk test. For normal data with multiple comparisons, unpaired one-way or two-way ANOVA (α = 0.05) was utilized. Post-hoc tests for multiple comparisons utilized the recommended multiple comparison test by Prism. For data without multiple comparisons, a two-tailed, unpaired Student’s *t* test (α = 0.05) was implemented. Homoscedasticity was evaluated using an F-test for single comparisons, with Bartlett’s and Brown-Forsythe test used for multiple comparisons; if values were heteroscedastic, Welch’s *t* test was utilized. Comparisons to percent of control were evaluated with a one-sample test with the Bonferroni-Holm correction (43). Lipidomics data were processed via univariate and multivariate analyses as mentioned above. Volcano plots utilized FDR = 1% (Benjamini, Krieger, Yekutieli method). NR12S ratiometric data were pre-processed for outliers using a conservative outlier test (ROUT = 1%).

## Results

### OW mammarenavirus infection is dependent on both exogenously supplied and endogenously produced lipids

**Fig. 1:**
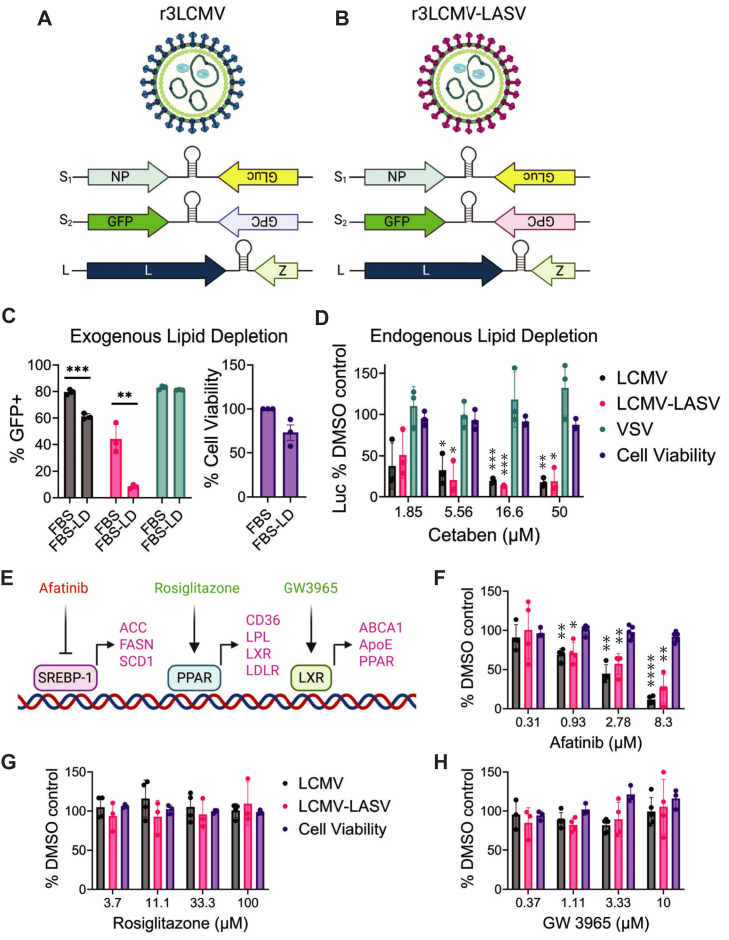
r3LCMV–LASV and r3LCMV are sensitive to both exogenous and endogenous lipid depletion. Genome schematic of r3LCMV (A) and r3LCMV-LASV (B) depicting where GLuc and GFP were added. C: VeroS cells were infected with r3LCMV (MOI 0.01), r3LCMV-LASV (MOI 0.01), or VSV-GFP (MOI 0.001) for 1 h, virus was removed, cells were washed, and media containing either normal FBS or lipid depleted FBS (FBS-LD) was added. The number of GFP positive cells were enumerated 48 h following r3LCMV (black) and r3LCMV-LASV (pink), while VSV (green) was harvest at 24 h. Each data point represents the percentage of GFP-positive cells from 10,000 live events; three independent trials were completed. Cell viability (purple) was compared at 48 h. Each data point represents the average of three 96-wells; three independent trials were completed. D: VeroS cells were infected with r3LCMV (MOI 0.01), r3LCMV-LASV (MOI 0.01), or VSV-NLucP (MOI 0.001) and treated with the indicated concentrations of cetaben. GLuc was measured 48 h following mammarenavirus infection and NLuci was measured 24 h following VSV-NLucP infection. Luciferase levels relative to DMSO control are shown. Each data point represents the relative luciferase levels from the average of three 96-wells. Two-way ANOVA was employed for statistical analysis; *P*≤ 0.05 (∗), *P*≤ 0.01 (∗∗), *P*≤ 0.001 (∗∗∗), *P*≤ 0.0001 (∗∗∗∗). E: Schematic of the regulatory pathways targeted by afatinib, rosiglitazone, and GW3965. F-H: Small molecule inhibition assay of each indicated drug performed on Vero cells. Data points represent the mean of triplicate wells, with three independent biological replicates. Student’s *t* test was employed to test for significance between DMSO control and treatment. *P*≤ 0.05 (∗), *P*≤ 0.01 (∗∗), *P*≤ 0.001 (∗∗∗), *P*≤ 0.0001 (∗∗∗∗).

Previous studies demonstrated successful arenavirus reproduction requires cholesterol during entry and fatty acids for proper protein localization (24, 27, 30). In cell culture and in vivo, cellular lipids can be made by de novo synthesis within the cell or scavenged through uptake of exogenous lipoproteins present in media or the extracellular milieu. To determine if mammarenavirus reproduction relies on the uptake of exogenous lipoproteins provided within the fetal bovine serum (FBS), we maintained cells in media supplemented with lipoprotein–depleted FBS (FBS-LD) or traditional FBS. Cells were infected with either a recombinant tri-segmented LCMV containing a duplication of the S segment that introduced both GFP and Gaussia luciferase (GLuc) reporter genes (r3LCMV) (Fig. 1A); or a r3LCMV where LCMV GPC was replaced with LASV GPC (r3LCMV-LASV) (Fig. 1B). Replication of these viruses is readily monitored by either GFP or GLuc expression. The number of GFP-positive infected cells was significantly reduced in cells maintained in the lipoprotein-depleted media (Fig. 1C), suggesting exogenous lipids facilitate optimal LCMV and LCMV-LASV reproduction. Lipoprotein depletion does not uniformly decrease all viral replication, as a recombinant vesicular stomatitis virus (VSV) expressing GFP was not affected, and cell viability was not significantly altered (Fig. 1C).

To determine if endogenously produced lipids are also important for replication, we infected cells in the presence of cetaben. Cetaben is a hypolipidemic compound known to induce peroxisome proliferation and subsequent β-oxidation of very-long-chain fatty acids (VLCFA); treatment with cetaben is associated with decreased levels of cholesterol, triglycerides, and cholesterol esters among other complex lipids (44, 45, 46). Cetaben significantly reduced luciferase production of both r3LCMV and r3LCMV-LASV without altering cell viability or VSV-NluciP luciferase production (Fig. 1D), suggesting optimal reproduction of LCMV might require VLCFAs, cholesterol, triglycerides, or other complex lipids degraded by peroxisome proliferation.

Cellular lipid synthesis and uptake pathways are highly regulated. Several cellular pathways lead to changes in transcription of genes involved in lipid synthesis and uptake, which are referred to as lipid regulatory elements (LRE) (Fig. 1E). To determine whether LREs such as sterol regulatory element-binding protein (SREBP), liver X receptors (LxRs), and peroxisome proliferator-activated receptors (PPARs) were consequential in LCMV replication, we measured the effect of various LRE inhibitors or agonists on viral replication. Inhibition via afatinib disrupts the ability of the cell to induce SREBP-1 cleavage through the PI3K/Akt/mTorC pathway, previously shown to restrict mammarenavirus infection (47, 48). Afatinib treatment reduced r3LCMV and r3LCMV-LASV replication in a dose-dependent manner, while agonists for PPARγ (rosiglitazone) and LxRs (GW 3965) did not alter viral GLuc expression (Fig. 1F–H).

### Mammarenavirus infection alters the cellular lipidome

**Fig. 2:**
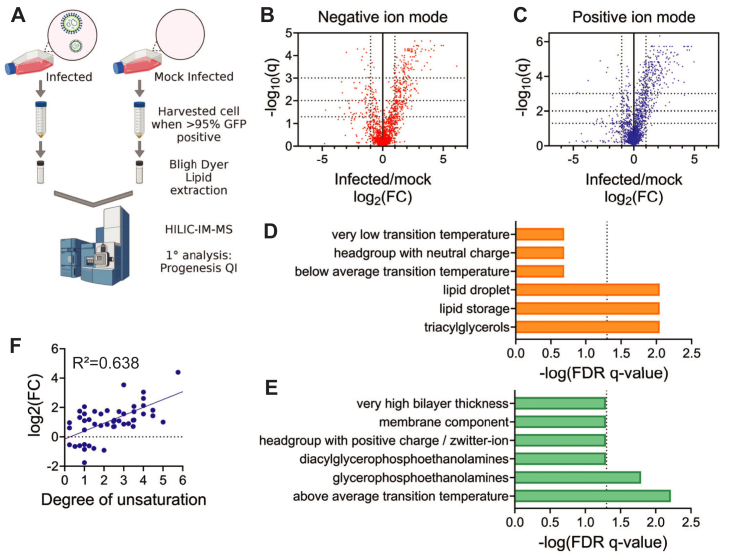
r3LCMV–LASV-infected VeroS cells exhibit altered lipidomes. A: Schematic of lipidomic process. Confluent VeroS cells were infected at 0.75 MOI with r3LCMV-LASV or mock (PBS); 72hpi, cells were harvested by scraping and subjected to Bligh and Dyer lipid extraction and HILIC-IM-MS and species calling by Progenesis QI. Volcano plots of negative (B) and positive (C) ion modes, comparing infected lipid abundance to mock. q-values calculated via multiple unpaired t-tests (Method: Benjamini, Krieger, Yekutieli, FDR = 1%). LIONWeb analysis of n = 54 lipids From PCA analysis; these indicate lipid characteristics/species that positively (D) and negatively (E) correlate with the infected cell lipidome. F: Increases in unsaturation of fatty acid chains during infection, linear regression.

r3LCMV and r3LCMV-LASV replication were dependent on both exogenous lipid availability and on endogenous lipid synthesis suggesting that mammarenavirus infection could influence host metabolism and alter the cellular lipidome. To monitor the effects of r3LCMV-LASV infection on the cellular lipidome, VeroS cells were infected and harvested at 72 hpi when 95% of cells were GFP positive. Lipids were extracted from both r3LCMV-LASV- and mock-infected cells using a modified Bligh-Dyer method and identified using hydrophilic-interaction liquid chromatography with ion mobility-mass spectrometry (HILIC-IM-MS) (Fig. 2A) (36). A vast number of lipids were altered due to viral infection (Fig. 2B, C, and PCA analysis identified 52 total lipid specie that contributed to the majority of the differences between infected and uninfected samples (supplemental Table S1). To determine how our lipidomics data related to chemical, biophysical and cell biology features, we processed the data through the lipid ontological analysis browser LIONWeb (Fig. 2D, E) (49). LIONWeb Analysis with two-tailed enrichment suggested that the features most positively associated with the r3LCMV-LASV infected cellular lipidome were triacylglycerols, lipid droplets, and lipid storage (Fig. 2D). The r3LCMV-LASV lipidomics were negatively associated with phosphatidylethanolamine (PE) and above average transition temperatures, suggesting a possible bias toward increased unsaturation among lipids in the infected cells (Fig. 2E). Bolstering this association, the lipidomic data suggests infection is positively associated with increased lipid unsaturation, especially for 4- and 6-degrees of unsaturation (Fig. 2F).

**Sup Fig 1:**
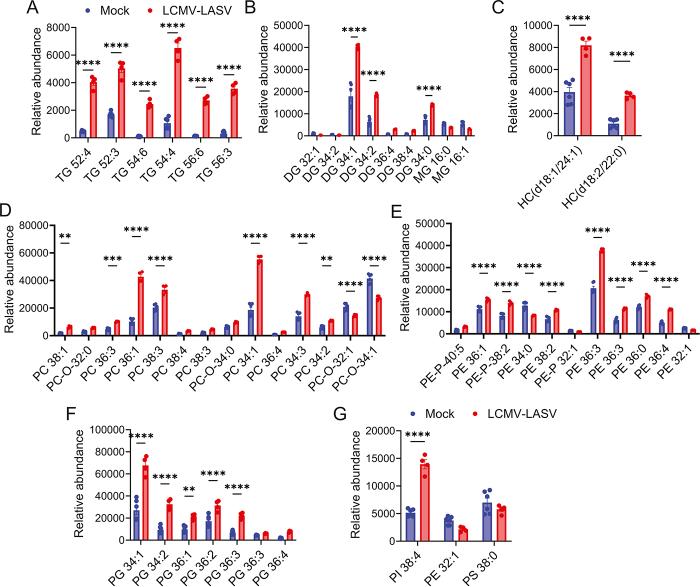
Lipid species are altered in r3LCMV-LASV infected VeroS cells. HILIC-IM-MS evaluated relative abundance of cellular lipids within r3LCMV-LASV infected (Red) and uninfected (Blue) cells. Positive-mode identified species: A) triacylglycerol (TG), B) diacylglycerol (DG), monoacylglycerol (MG), C) hexosylceramides (HC), phosphatidylinositol (PI), and phosphatidylserine (PS). D) phosphatidylcholine (PC), phosphatidylcholine with an ether-linked (plasmanyl) acyl chain (PC-O), E) phosphotidylethanolamine (PE) and phosphatidyl ethanolamine with a vinyl-ether linked (plasmenyl) acyl chain (PE-P). Negative-mode identified species: F) phosphatidylglycerol (PG) and G) phosphatidylinositol (PI), and phosphatidylserine (PS), and PE. (PG). Statistics performed via student’s t-test. *P* ≤ 0.05 (∗), *P* ≤ 0.01 (∗∗), *P* ≤ 0.001 (∗∗∗), *P* ≤ 0.0001 (∗∗∗∗).

Infection resulted in a significant increase in multiple species of triacylglycerol (TG) (supplemental Fig. S1A). Several species of diacylglycerol (DG) (supplemental Fig. S1B), two species of hexosylceramide (HexCer) and a phosphatidylinositol (PI) (supplemental Fig. S1C), phosphatidylcholines (PC) (supplemental Fig. S1D), phosphatidylethanolamine (PE) (supplemental Fig. S1E), and phosphatidylglycerol (PG) (supplemental Fig. S1F) were enriched during infection. Two species of monoacylglycerol (MG), one species of phosphatidylethanolamine (PE) and phosphatidylserine (PS), and two ether-linked PC were decreased in infected cells (supplemental Fig. S1G). The substantial increase in TG and DAG, without corresponding decreases in other lipid species, suggests infection induces de novo fatty acid synthesis.

### Lipid droplet formation is not critical for mammarenaviral infection

**Fig. 3:**
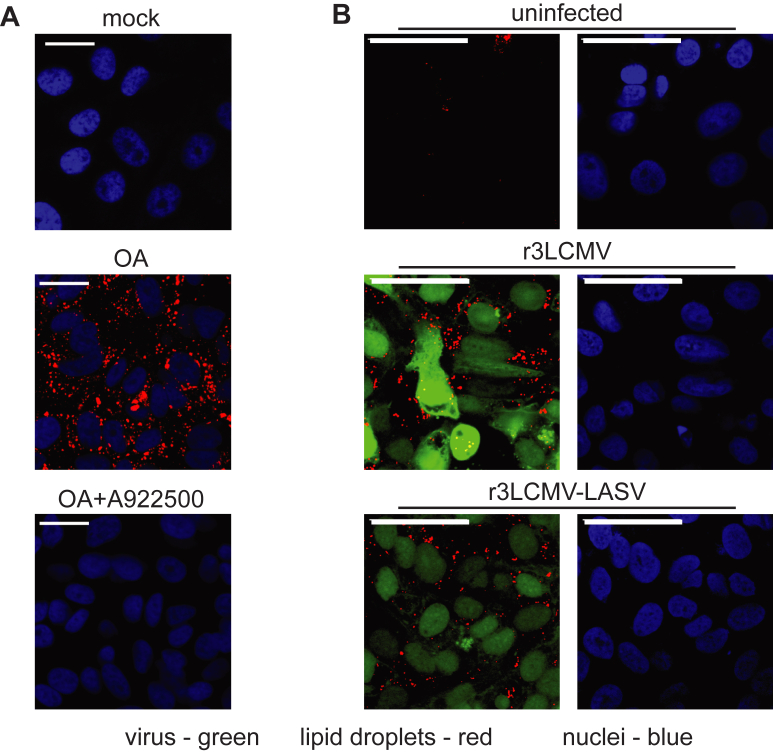
r3LCMV-LASV and r3LCMV infection of VeroS cells is associated with increased lipid droplets. A: VeroS cells were treated with the following conditions: mock, DMSO (0.1% v/v) + EtOH:BSA (100 μM:0.02 μM); OA, DMSO (0.1% v/v) + OA:BSA (100 μM:0.02 μM); OA + A922500, A922500 50 μM + OA:BSA (100 μM:0.02 μM). Cells were harvested 24 h post-treatment, fixed, treated with Hoechst 33,342 (1 μg/ml) and HCS LipidTox Red Neutral Lipid Stain (1:1,000X) and imaged using confocal (600X magnification). Scale bars indicated 50 μm. B: VeroS cells were either mock-infected or infected with r3LCMV (0.01 MOI) or r3LCMV-LASV (0.01 MOI), and harvested at 48 hpi following infection. Cells were fixed in 3.7%v/v formalin, stained with Hoechst 33,342 (1 μg/ml) and HCS LipidTox Red Neutral Lipid Stain (1:1,000X), and imaged using the confocal at 600X magnification with negligible digital zoom. Scale bars indicated 50 μm.

Lipidomic analysis identified a significant increase in triglycerides (TGs) after infection (supplemental Fig. S1A). Cells store excess toxic fatty acids in the form of TG in lipid droplets (LD), which can be visualized in cells using LipidTOX deep red stain. While untreated VeroS cells displayed a few random LDs (Fig. 3A), cells treated with oleic acid (OA), a fatty acid, were found to have increased red puncta throughout, indicating formation of LDs (Fig. 3A). Addition of a diacylglycerol acyltransferase (DGAT) inhibitor, A922500, which prevents the incorporation of oleic acid into TGs, blocked the formation of LD, demonstrating the staining was detecting TGs condensed in LD (Fig. 3A). VeroS cells infected with either r3LCMV or r3LCMV-LASV contained more LDs than uninfected cells (Fig. 3B), confirming these viruses increase the level of TG during infection.

**Sup Fig 2:**
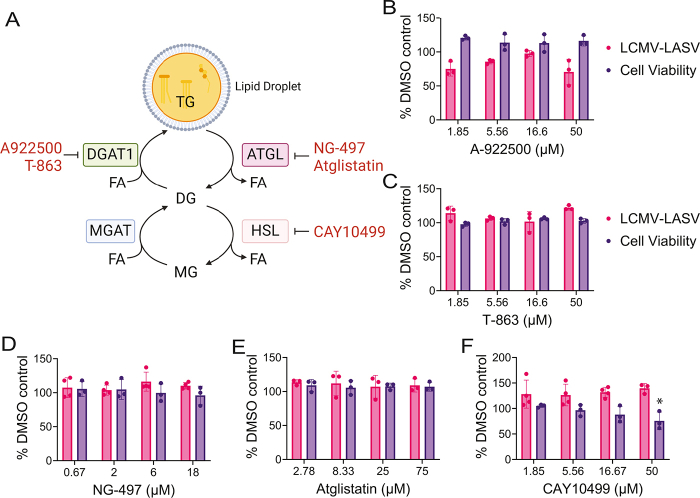
r3LCMV-LASV is not sensitive to inhibitors of neutral lipid (MG, DG, and TG) catabolism and anabolism. A) Catabolic and anabolic pathways for neutral lipid metabolism with pharmacologic inhibitors of the indicated enzymes. B-F) Small molecule-virus inhibition assays (pink) paired with cytotoxicity assays (purple). For the virus inhibition assays, confluent Vero cells were infected at a low MOI (0.01) with r3LCMV-LASV and treated with the indicated compound at multiple indicated concentration, or equivalent amount of DMSO. Cells were harvested at 48 hpi using the Pierce Gaussia Luciferase kit. To measure cytotoxicity, confluent Vero cells were treated with the indicated compounds (or DMSO) and harvested at 48 h with Cell Titer-Glo. Data is representative of at least 3 independent, biological replicates of triplicates.

TG is synthesized by esterification of acyl-CoA and DG, catalyzed via DGAT1, and can be degraded by adipose triglyceride lipase (ATGL) back into DG (supplemental Fig. S2A). To determine whether high TG levels are necessary for arenavirus replication, we infected cells in the presence of DGAT1 inhibitors A-922500 or T-863 (50, 51) or the ATGL inhibitors NG-497 or atglistatin (52, 53). While A-922500 decreased LD formation after oleic acid treatment (Fig. 3A), virus GLuc reporter activity was unaffected (supplemental Fig. S2B). Similarly, T-863 did not affect r3LCMV-LASV infection (supplemental Fig. S2C). Blocking the degradation of TG back into DG with ATGL inhibitors, which would increase TG levels, also did not alter r3LCMV-LASV (supplemental Fig. S2D, E). Furthermore, blocking lipolysis of DG to MG via CAY10499 inhibition of hormone sensitive lipase (HSL) had no effect on viral replication (supplemental Fig. S2F). These results indicate that TG levels, and functionally lipid droplet formation, are not critical for r3LCMV-LASV replication.

### De novo fatty acid synthesis is required for mammarenavirus infection

**Fig. 4:**
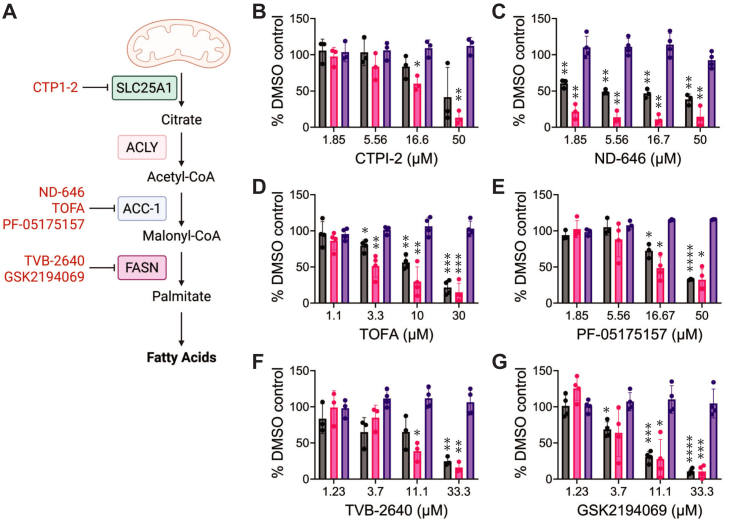
Inhibition of multiple enzymes within the fatty acid biosynthetic pathway limits r3LCMV-LASV and r3LCMV reporter gene levels. A: Anabolism of fatty acid synthesis: (1) solute carrier protein 25A1 (SLC25A1) enables export of citrate (2) ATP-citrate lyase cleaves citrate into acetyl-CoA and oxaloacetate, and coordinates CoA to form acetyl-CoA 3) acetyl-CoA carboxylase yields the three carbon malonyl-CoA, that is used as the template for 4) FASN to synthesize palmitate over 8 cycles of synthesis, with acetyl-CoA as the two-carbon donor. B-G: Small molecule-virus inhibition assays of r3LCMV-LASV (pink) and r3LCMV (gray) paired with cytotoxicity assays (purple). Vero cells were infected with r3LCMV and r3LCMV-LASV at a low MOI (<0.01) and treated with the indicated compounds at multiple concentrations, or equivalent levels of DMSO. Luciferase levels were measured at 48 h using the Pierce Gaussia luciferase kit. Data is representative of at least 3 independent, biological replicates of triplicates. One sample *t* test with Bonferroni-Holm correction. *P*≤ 0.05 (∗), *P*≤ 0.01 (∗∗), *P*≤ 0.001 (∗∗∗), *P*≤ 0.0001 (∗∗∗∗).

An excess of TGs and lipid droplets within infected cells, though not critical for r3LCMV-LASV replication, suggests infection leads to an increase in fatty acid levels. Fatty acids (FA) can be obtained through either hydrolysis of esterified lipids (TG, DG, phospholipids, etc), or through de novo synthesis via fatty acid synthase (FASN). De novo FA synthesis occurs via a multistep process (1) the conversion of exported citrate to acetyl-CoA by ATP-citrate lyase (ACLY), (2) production of malonyl-CoA by acetyl-CoA carboxylase (ACC), and (3) polymerization of acetyl-CoA (C2) to malonyl-CoA (C3) mediated by FASN; after seven cycles of FASN-mediated polymerization, palmitate (C16:0) will be produced (Fig. 4A). To determine if fatty acid production is required for efficient mammarenavirus replication, we used several inhibitors of the fatty acid synthesis pathway (Fig. 4A). Inhibitors of SLC25A1, ACLY, ACC1/2, and FASN significantly decreased r3LCMV and r3LCMV-LASV replication (Fig. 4B–G), further suggesting that fatty acids are required for replication.

**Fig. 5:**
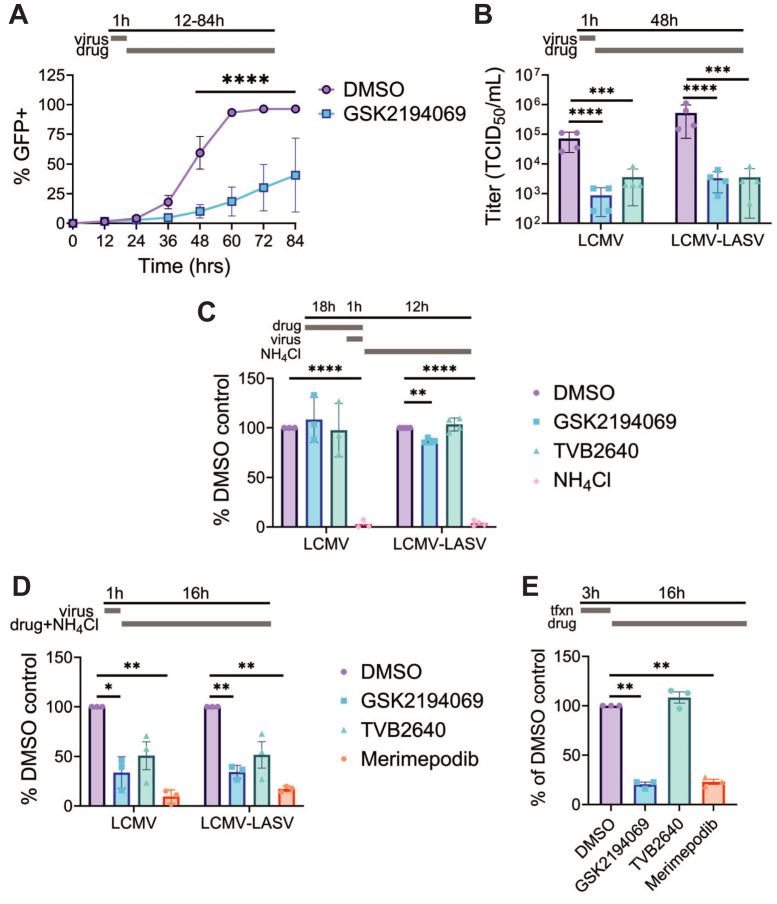
FASN inhibition inhibits multi-step infection, replication, infectious particle production, and budding of r3LCMV-LASV and r3LCMV. A: Multi-step growth curve of r3LCMV-LASV in VeroS cells, treated with GSK2194069 (33 μM) or DMSO. VeroS cells were briefly infected with either virus at a low MOI (0.001), virus was removed, treatment was applied, cells were harvested at the indicated time-points and analyzed via flow-cytometry. B: Production of infectious particles at 48 hpi from infected (0.01 MOI) VeroS cells treated DMSO, GSK2194069 (33 μM), or TVB-2640 (33 μM). C: Entry assay of either virus into VeroS cells. VeroS cells were pre-treated for 18 h with DMSO, GSK2194069 (33 μM), TVB-2640 (33 μM), or NH_4_Cl (10 mM) prior to application of the indicated virus (MOI = 3). After 1.5 h of infection/treatment, infection/treatment was removed and replaced with media containing NH_4_Cl (10 mM), to prevent further entry. Cells were harvested for flow cytometry at 16–18 hpi. D: Luciferase-levels within a single step of infection. VeroS cells were infected with the indicated virus at an MOI of 0.5 for 1.5 h, washed, and treated with the indicated compounds (0.1% v/v DMSO, GSK2194069 (33 μM), TVB-2640 (33 μM), or merimepodib (12.5 μM). Supernatants were harvested at 16 hpi (approx. 50% GFP+), and secreted Gaussia luciferase levels were evaluated with the Pierce Gaussia Luciferase assay kit. E: Effects of drugs on expression of NLuci-PEST from transfected plasmid; cells were transfected for 4 h, followed by removal of transfection reagent and replacement with DMEM + 5% FBS + 0.1% v/v DMSO, GSK2194069 (33 μM), TVB2640 (11 μM), or merimepodib (12.5 μM). Luminescence measured at 16 h post-treatment. *P*≤ 0.05 (∗), *P*≤ 0.01 (∗∗), *P*≤ 0.001 (∗∗∗), *P*≤ 0.0001 (∗∗∗∗).

To determine when fatty acids are needed during the replication cycle, we completed follow up studies with two FASN inhibitors, TVB-2640 and GSK2194069, each known to bind the ketoacyl reductase domain of FASN (54, 55). First, cells were infected at a low MOI (0.01) for 1 h, and the compounds were added after the viral inoculum was removed. Cells were monitored every 12 h to determine if FASN inhibition alters arenaviral spread. In vehicle-treated cells, the entire cell population was infected at 60 h post infection, while only 20% were infected in the presence of GSK2194069 (Fig. 5A). TVB-2640 and GSK2194068 treatment both reduced viral titer by > 25-fold 48 h post infection (Fig. 5B). To determine if FASN inhibition directly affects arenavirus entry, cells were treated with GSK2194069 or TVB-2640 for 18 h to decrease cellular fatty acid levels. Cells were then infected for one hour, at which time the inoculum and drugs were removed, and media containing ammonium chloride (NH_4_Cl) was added to inhibit subsequent entry events. Neither GSK2194069 nor TVB-2640 treatment altered the number of virus-infected cells, suggesting FASN activity is not required for viral entry (Fig. 5C). Next, to determine if FASN activity is needed for viral transcription/translation, cells were infected for one hour, treated with inhibitors, and monitored for viral reporter protein production. While merimepodib treatment, a compound previously shown to block several steps in the arenaviral lifecycle (56), reduced GLuc production to 15% of the DMSO control, GSK2194069 decreased levels to 35%, and TVB-2640 reduced luciferase levels to 50% (Fig. 5D), suggesting blocking FASN decreases viral transcription or translation. To ensure that the decrease in luciferase levels is not due to an overall, non-specific inhibition of transcription and protein synthesis, we evaluated the effect of inhibitor treatment on expression of plasmid-driven nano-luciferase levels (Fig. 5E). Luciferase-levels within TVB-2640 treated cells were similar to vehicle (DMSO); however, both GSK2194069 and merimepodib were found to reduce plasmid driven luciferase levels, suggesting they both affect cellular transcription/translation and are not viral specific.

### FASN inhibition reduces mammarenavirus particle production without major changes to Z localization

**Fig. 6:**
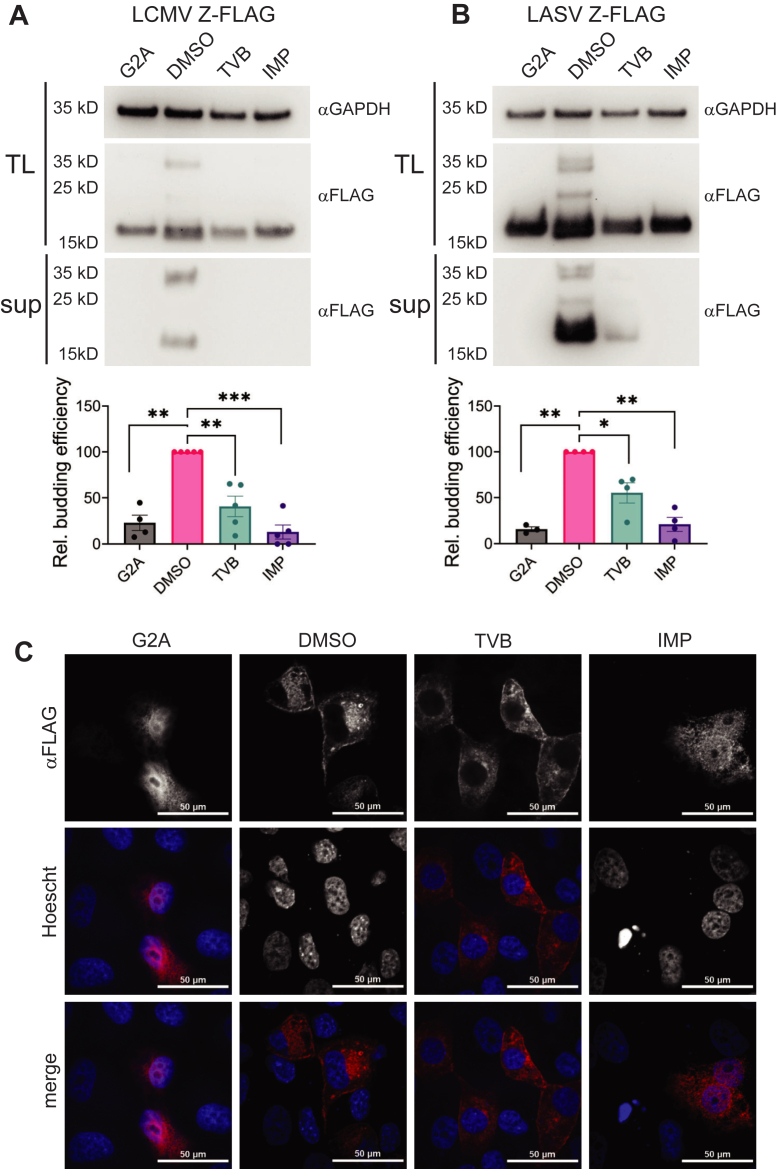
Effects of FASN inhibition in 293T cells on LCMV Z and LASV Z budding and localization. Immunoblot of LCMV (A) and LASV (B) Z-FLAG and Z-G2A-FLAG transfected 293T cell supernatant (sup) and total cell lysate (TL) treated with DMSO (0.1v/v%), TVB-2640 (33 μM), or IMP (0.1 μM). Quantification of additional experiments shown below; relative budding efficiency compares the sup/Tl levels in the different treatment groups to the DMSO control. One sample *t* test with Holm-Sidak multiple comparison test, *P*≤ 0.05 (∗), *P*≤ 0.01 (∗∗), *P*≤ 0.001 (∗∗∗), *P*≤ 0.0001 (∗∗∗∗). C: Confocal images (600X) of VeroS cells transfected with LASV-Z-FLAG or Z-G2A-FLAG (Red) and treated DMSO (0.1%v/v), TVB2640 (33 μM), or IMP (1.0 μM), and stained with Hoechst 33342 (Blue).

**Sup Fig 3:**
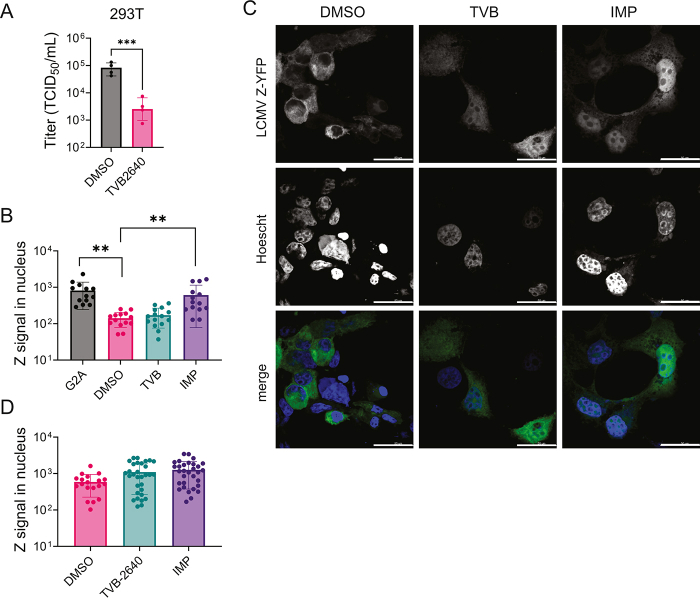
Effects of FASN inhibition in 293T cells on LCMV Z and LASV Z budding and localization A) r3LCMV-LASV infectious viral titer is inhibited by TVB-2640 treatment in 293T cells (0.01 MOI, 48 h.p.i.); student’s t-test, *P* ≤ 0.001 (∗∗∗). (B) Quantification of confocal data presented in Figure 6C; n = 15 cells per treatment group within one replicate. One-Way ANOVA, *P* ≤ 0.01 (∗∗). (C) Confocal images (600X) of VeroS cells transfected with LCMV-Z-HA-YFP and treated with DMSO (0.1%v/v), TVB-2640 (33μM), and IMP-1088 (1μM). (B) Quantification of confocal data presented in (C); n = 18-31 cells per treatment group within one replicate.

TVB-2640 reduced viral titers without altering cellular transcription and translation or viral entry, suggesting late steps in viral replication, such as budding, may be inhibited. To investigate the effect of FASN inhibition on arenavirus Z budding, we monitored production of virus-like particles (VLP) produced by LCMV Z or LASV Z within transfected 293T cells; note inhibition of LCMV-LASV by TVB-2640 was reproduced in 293T cells (supplemental Fig. S3A). Budding mediated by mammarenavirus Z is known to require NMT-mediated myristoylation of the protein; therefore, we included a myristoylation-deficient Z-G2A mutant for comparison along with an NMT inhibitor, IMP-1088 (57). Cells transfected with wild-type Z were treated with DMSO, TVB-2640, or IMP-1088; supernatants and cell lysates were collected 24 h following treatment. As expected, the Z-G2A mutants as well as IMP-1088 treatment failed to produce VLPs in the supernatant (Fig. 6A, B). Treatment with TVB-2640 had a similar effect, decreasing budding of both LCMV Z and LASV Z. Furthermore, intracellular Z-homodimer bands at ∼30 kDa were absent or decreased in G2A mutants, IMP-1088, and TVB-2640 treatment groups, suggesting that myristoylation and FASN inhibition might each impair Z oligomerization.

To discern whether the decrease in budding and oligomerization could be due to changes in the localization of Z by FASN-inhibition, we used immunofluorescent confocal microscopy to monitor LASV Z. In both DMSO and TVB-2640 treatment groups, LASV Z was primarily found at the plasma membrane and organellar membrane, with a distinct void of immunofluorescence within the nucleus (Fig. 6C and supplemental Fig. S3B). IMP-1088-treated LASV Z and LASV Z-G2A each displayed diffuse cytoplasmic distribution and immunofluorescence within the nucleus. Together, these data suggest that TVB-2640 decreases budding without significant changes to LASV Z localization, suggesting that depletion of FASN-derived acyl groups is not strong enough to demonstrate noticeable localization changes. While LASV Z localization was not markedly altered by TVB-2640, LCMV Z-YFP was present in the nucleus when treated with either IMP-1088 or TVB-2640 (supplemental Fig. S3B, D), supporting potential increased sensitivity to TVB-2640 as seen in (Fig. 6C).

### Oleic acid restores mammarenaviral titers repressed by FASN inhibition

**Fig. 7:**
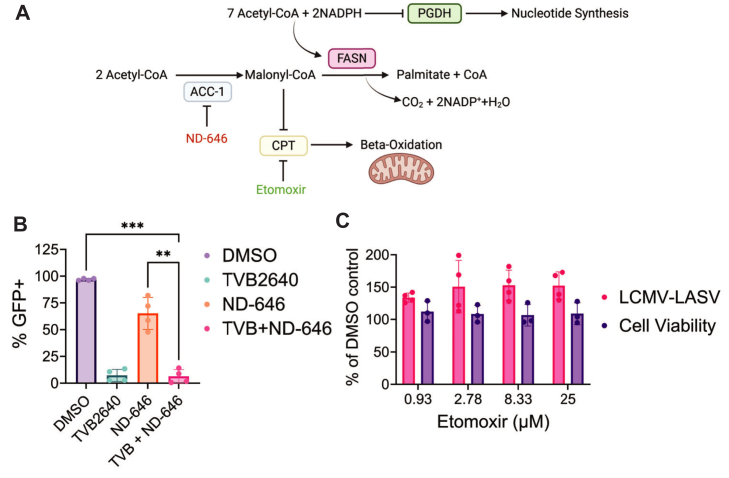
Side effects of FASN inhibition have no effect on r3LCMV-LASV. A: Diagram of chemical reaction facilitated by FASN. B: Depletion of malonyl-CoA by ND-646 co-treatment with TVB-2640 treatment does not relieve TVB-2640-associated inhibition of r3LCMV-LASV (VeroS cells, 0.01 MOI, 48 hpi); DMSO (0.1% v/v), TVB-2640 (33 μM), ND-646 (5 μM). One-Way ANOVA with Dunnett’s T3 Multiple Comparison Test, *P*≤ 0.01 (∗∗), *P*≤ 0.001 (∗∗∗). C: Inhibition of beta-oxidation by etomoxir does not inhibit r3LCMV-LASV reporter gene activity (pink); etomoxir is not cytotoxic (purple) in Vero cells.

FASN inhibition has three direct consequences within cells: (1) an increase in malonyl-CoA, the starting substrate, (2) an increase in NADPH, and (3) a decrease in palmitate synthesis (Fig. 7A). Each of these effects could be responsible for limiting r3LCMV and r3LCMV-LASV replication. Malonyl-CoA is a repressor of carnitine palmitoyltransferase I (CPTI) (58). Inhibition of CPTI decreases lipid beta-oxidation and inhibits acylation (59). However, when FASN and the synthesis enzyme for malonyl-CoA, ACC1/2, are both inhibited by a combination treatment of TVB-2640 and ND-646 (60), there is a compounding effect (Fig. 7B), suggesting that the inhibition is not a result of increased malonyl-CoA concentration blocking CPTI. Furthermore, independent inhibition of CPTI by etomoxir (61) has no effect on r3LCMV-LASV or r3LCMV replication (Fig. 7C). Both these results support the conclusion that inhibition of virus by FASN inhibition is not due to acylation or beta-oxidation inhibition secondary to increases in malonyl-CoA.

**Fig. 8:**
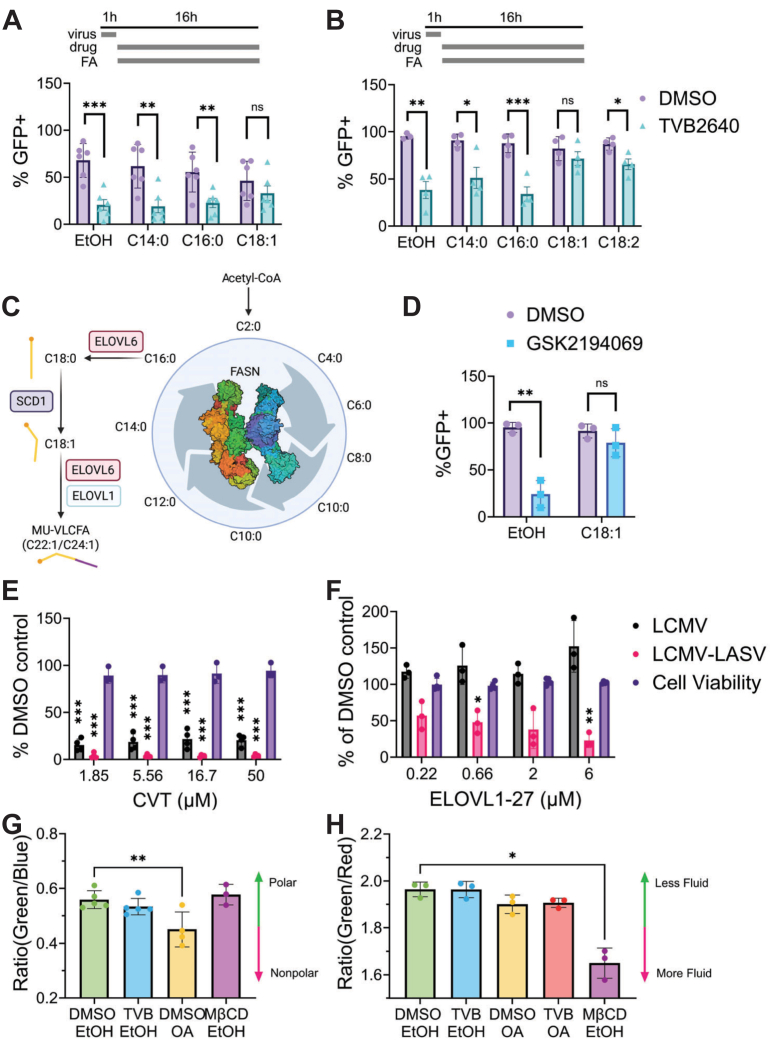
Addback of C18:1 restores r3LCMV-LASV infection. Addback of EtOH, C14:0, C16:0, C18:1, or C18:2-BSA during DMSO (0.1%v/v) or TVB-2640 (33 μM) treatment of VeroS cells infected with (A) r3LCMV or (B) r3LCMV-LASV (0.01 MOI, 48hpi). C: Diagram of FASN biosynthesis and downstream products. D: C18:1 rescues GSK2194069-associated inhibition of r3LCMV-LASV in VeroS cells (0.01 MOI, 48hpi). E: CVT-12012 inhibits r3LCMV (black) and r3LCMV-LASV (red) viral reporter gene levels in a dose-responsive manner in Vero cells, without cytotoxicity (grey). (0.01 MOI, 48hpi), Student’s unpaired *t* test. F: ELOVL1-27 treatment inhibits r3LCMV-LASV but not r3LCMV (VeroS cells, 0.01 MOI, 48hpi). One sample *t* test with Bonferonni-Holm correction. G: Laurdan-assessed VeroS cell lipid polarity is not altered by TVB-2640 (33 μM) or MbCD (5 mM) relative to DMSO (48hpt), but is altered by oleic acid addition (200 μM). MbCD (5 mM) treatment significantly increases membrane fluidity. H: NR12S-assessed membrane fluidity is not altered by TVB-2640-treatment relative to DMSO-treatment (48hpt). MbCD (5 mM) treatment significantly increases membrane fluidity. One-Way ANOVA with Dunnett’s T3 Multiple Comparison test, *P*≤ 0.05 (∗), *P*≤ 0.01 (∗∗), *P*≤ 0.001 (∗∗∗), *P*≤ 0.0001 (∗∗∗∗).

Increasing levels of NADPH due to FASN inhibition will inhibit phosphogluconate dehydrogenase (PGDH) (Fig. 7A), the oxidoreductase enzyme that oxidizes 6-phosphogluconate into ribulose-5-phosphate, a precursor for the phospho-carbohydrate backbone of RNA and DNA (62). The increase in NADPH levels will reduce PGDH activity, and the subsequent decrease in pentose phosphate activity could account for the inhibition of r3LCMV reproduction. Other nucleotide synthesis inhibitors, such as the IMPDH inhibitor merimepodib, an inhibitor of purine de novo synthesis, reduces LASV titers in vitro (56). To test whether r3LCMV-LASV is inhibited by FASN through increased NADPH levels or through depletion of the fatty acids produced by FASN, we applied the BSA-conjugated fatty acids myristic acid (C14:0), palmitic acid (C16:0), oleic acid (C18:1), and linoleic acid (C18:2) during treatment with vehicle (DMSO) or TVB-2640. r3LCMV infection level during TVB-2640 treatment was mildly rescued by oleic acid (Fig. 8A) and oleic acid strongly rescued r3LCMV-LASV (Fig. 8B). Surprisingly, addition of the primary fatty acid produced from FASN activity, palmitic acid, did not restore r3LCMV-LASV or r3LCMV infection during FASN inhibition, nor did supplementation with myristic acid (Fig. 8A, B); addback of linoleic acid rescues, but does not fully restore r3LCMV-LASV (Fig. 8B). Oleic acid also rescued GSK2194069-mediated inhibition of r3LCMV-LASV (Fig. 8D), supporting the finding that FASN inhibition is likely due to depletion of unsaturated fatty acids, not increases in NADPH. Furthermore, application of CVT-12012, an inhibitor of stearoyl-CoA desaturase (SCD), the enzyme that catalyzes the conversion of stearic acid to oleic acid (Fig. 8C), reduces reporter gene activity of r3LCMV and r3LCMV-LASV within Vero cells (Fig. 8E). C18:1 forms the substrate upon which monounsaturated, very long-chain fatty acids (MU-VLCFA) such as C20:1, C22:1, and C24:1 are produced via ELOVL1 elongation (Fig. 8C) (63). We tested whether ELOVL1-27, an inhibitor of ELOVL1, inhibits r3LCMV and r3LCMV-LASV reporter gene production. r3LCMV-LASV, but not r3LCMV infection, was inhibited by ELOVL1-27 inhibition, but in general, inhibition was marginal, suggesting that elongation of C18:1 is not critical for arenavirus infection (Fig. 8F).

Because we observe that budding is impacted by FASN inhibition, we hypothesized that altered membrane fluidity might be responsible for the observed FASN-dependence of arenaviruses. We predicted that FASN inhibition would increase the lipid order of the cell membrane, and that oleic acid’s observed rescue effect is due to downstream integration into membrane phospholipids and subsequent increased membrane fluidity. To measure alterations in the intracellular lipid environment, we performed Laurdan staining of vehicle or treated cells and assessed their ratiometric shift by flow cytometry. While our control, oleic acid, significantly increased the non-polar environment within the cell, TVB-2640 did not alter the intracellular lipid environment, nor did methyl-beta-cyclodextrin (MβCD), which will strip plasma membrane cholesterol and increase plasma membrane fluidity (Fig. 8G). To specifically discern alterations in the plasma membrane lipid environment, we utilized NR12S, and found that while MβCD significantly increases plasma membrane fluidity, neither TVB-2640 nor OA altered plasma membrane lipid order (Fig. 8H).

### FASN dependence is a feature of multiple mammarenaviruses in disease-relevant cell lines

**Fig. 9:**
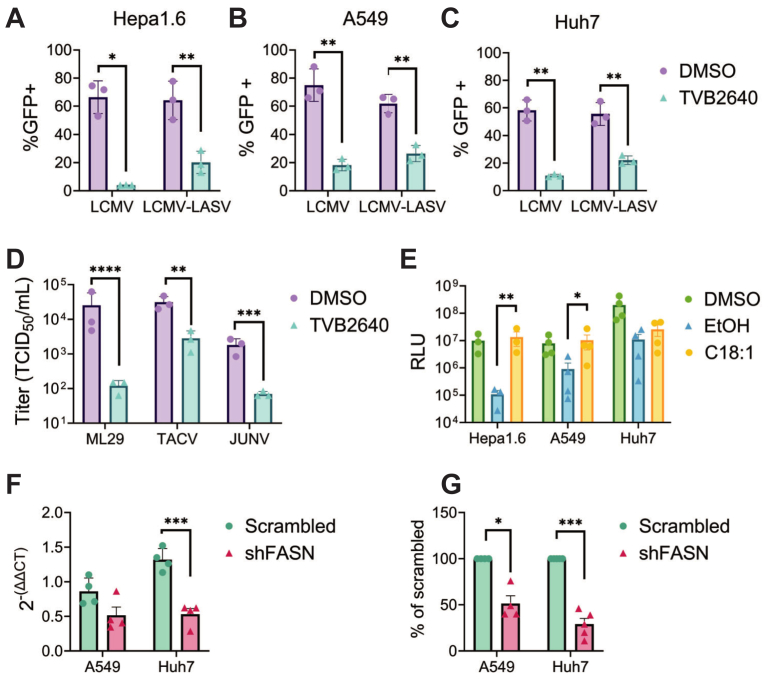
Mammarenaviruses depend on FASN in multiple cell lines. A–C: TVB-2640 treatment of (A) Hepa1-6, (B) A549, and (C) Huh7 cells reduces the number of r3LCMV and r3LCMV-LASV infected cells at 48 hpi (0.01 MOI for A549 and Hepa1-6 0.001 MOI for Huh7, 48hpi); Student’s or Welch’s unpaired *t* test (D) TVB-2640 (33 μM) treatment of VeroS cells decreases ML29, TACV, and JUNV infectious particle production (0.01 MOI, 48h.p.i). Two-Way ANOVA of ln-transformed data, Fisher’s LSD. E: Oleic acid rescues r3LCMV-LASV infection (0.01 MOI, 48 h) during TVB-2640 treatment of A549 and Hepa1-6 cells, but not Huh7 cell; Welch’s *t* test, *P*≤ 0.05 (∗), *P*≤ 0.01 (∗∗), *P*≤ 0.001 (∗∗∗), *P*≤ 0.0001 (∗∗∗∗). F) Knockdown of FASN in Huh7 and A549 cells by shRNA against FASN decreases FASN mRNA transcripts relative to “scrambled” shRNA, as evaluated by RT-qPCR and Delta-Delta Ct analysis. G: Knockdown of FASN in Huh7 and A549 decreases reporter gene levels in a multistep infection with r3LCMV-LASV (0.01 MOI for A549, 0.001 MOI in Huh7 cells, 48hpi).

**Sup Fig 4:**
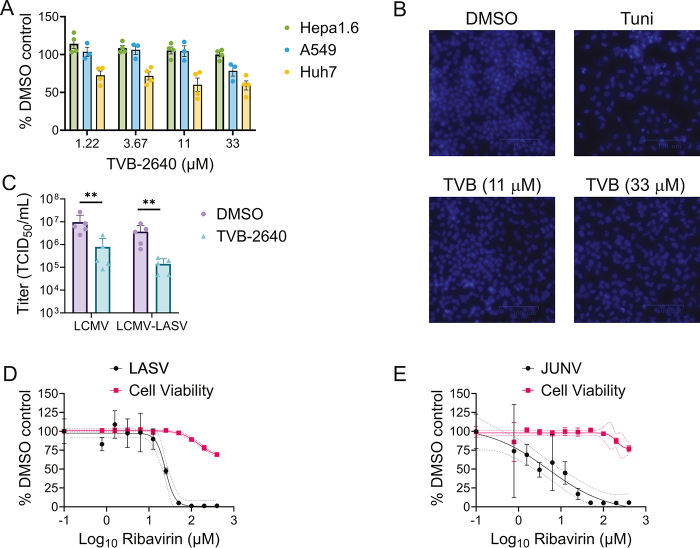
Cell toxicity controls. A) Cytotoxicity of DMSO and TVB2640 in Hepa1-6, A549, and Huh7 cells, as assessed by CellTiter-Glo. B) Effect of TVB-2640 (33 and 11μM) on Huh7 cell proliferation over 48hrs, compared to negative (DMSO) and positive (Tunicamycin, 1μM) controls for cell proliferation; Hoechst 33342 staining C) TVB-treatment of Hepa1-6 cells decreases r3LCMV-LASV and r3LCMV infectious particle production, statistics: unpaired t-test of ln-transformed data, *P* ≤ 0.01 (∗∗). D, E: Effects of ribavirin on D) r3LASV and E) r3JUNV infection (black) in A549 cells and A549 cell viability (pink).

To determine whether the FASN-dependence of r3LCMV-LASV and r3LCMV is conserved in disease-relevant cell lines such as hepatocytes and airway epithelial cells, human and mouse hepatocarcinoma cell lines (Huh7 and Hepa1-6), and lung carcinoma cell (A549) were utilized. Subtoxic concentrations of TVB-2640 (supplemental Fig. S4A), inhibited r3LCMV and r3LCMV-LASV spread in Hepa1.6 and A549 cell lines (Fig. 9A–C). Huh7 cells treated with TVB-2640 contained reduced levels of cellular ATP (supplemental Fig. S4A), though cells morphologically appear broadly unaffected; confocal images indicate decreased cell density relative to DMSO (supplemental Fig. S4B), implying TVB-2640 reduces Huh7 proliferation, similar to our control for cell proliferation, tunicamycin. In addition to the decrease of viral spread seen in Fig. 9A, TVB-2640 treatment in Hepa1-6 cells highly inhibits infectious viral titer (supplemental Fig. S4C) to a similar degree seen in VeroS cells (Fig. 5B).

Both r3LCMV and r3LCMV-LASV rely on the LCMV Z protein for budding and particle production. To determine if additional mammarenaviruses are sensitive to FASN-inhibition, we tested another OW mammarenavirus: ML29, a reassortant virus containing the NP and GPC from LASV and the Z and L from Mopeia virus (MOPV) (64). We also tested the NW mammarenaviruses Tacaribe virus (TACV) and the Candid 1 vaccine strain of Junin virus (JUNV). TVB-2640 reduced ML29 titers by 100-fold in VeroS cells while reducing TACV and JUNV titers more than 10-fold (Fig. 9D), suggesting FASN activity is needed across the mammarenavirus family. We also found that oleic-acid mediated rescue of r3LCMV-LASV infection in TVB-2640-treated cells is conserved within Hepa1-6 and A549 cells, but not Huh7 cells (Fig. 9E).

To demonstrate mammarenaviruses dependence on fatty acids without the use of small molecules, we transduced cells with lentiviral particles that produce either a scrambled or FASN target short hairpin RNA (shRNA). The shRNA modestly reduced FASN transcript levels in A549 while the knockdown was more efficient in the Huh7 cells (Fig. 9E). Viral produced luciferase levels were reduced in both A549 and Huh7 cells when FASN was knocked-down (Fig. 9G), further supporting efficient mammarenavirus replication requires active production of fatty acids.

**Fig. 10:**
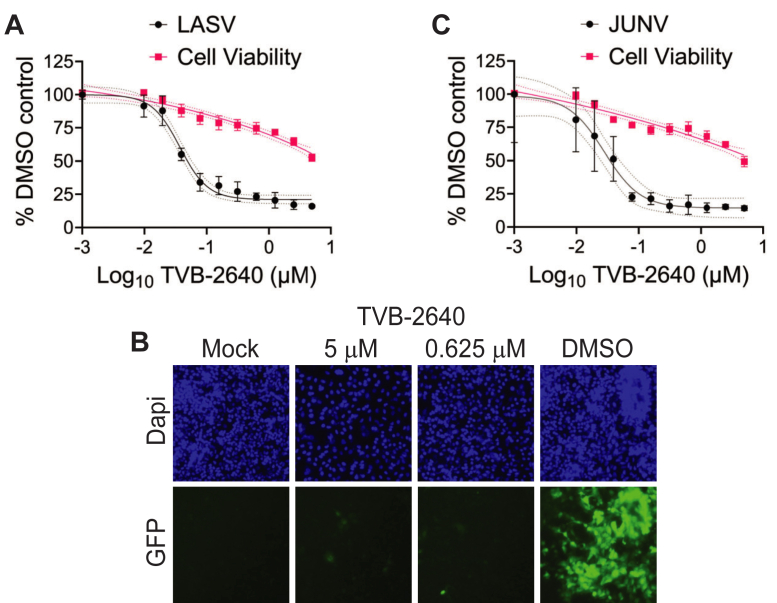
TVB-2640 inhibits pathogenic strains of mammarenaviruses. A: TVB-2640 treatment inhibits r3LASV (Josiah strain; IC50 = 37.02 ± 6.11 nM) and (B) r3JUNV (Romero strain; IC50 = 27.92 ± 10.44) infection in A549 cells at 72 and 96 h.p.i., respectively; technical quadruplicates, data representative of three independent trials.

Finally, we determined if TVB-2640 could limit replication of authentic Lassa and Junin viruses. Dose-response curves to TVB-2640 treatment of r3LASV-Josiah and r3JUNV-Romero infection in A549 cells in a BSL4 setting further suggests that highly pathogenic arenaviruses are sensitive to FASN inhibition at sub-cytotoxic concentrations (Fig. 10A–C), comparable to observed inhibition with the antiviral drug ribavirin (supplemental Fig. S4D, E).

## Discussion

The findings from this study illuminate the intricate relationship between mammarenavirus infection and host lipid metabolism. Both exogenous lipid uptake and endogenous lipid synthesis were crucial for the replication of r3LCMV and r3LCMV-LASV. Although the dependence on exogenous and endogenous lipids, as well as their regulation, could be dismissed as an in vitro artifact, the restriction of lipogenesis is a well-established interferon response (65, 66, 67, 68, 69, 70). This suggests that, in vivo, mammarenavirus control may involve limiting both endogenous lipid synthesis and exogenous lipid transport.

Pathological studies of LASV and LCMV-infected liver tissues show hepatic steatosis and increased lipid droplets, suggesting our in vitro model captures aspects of in vivo infection (71, 72, 73, 74). Consistently, lipid droplet induction is a common response to viral infection across multiple viruses (66, 75, 76, 77, 78). However, blocking lipid droplet formation did not affect r3LCMV or r3LCMV-LASV infection, indicating that lipid accumulation is likely a downstream effect of increased FASN activity or cellular stress. Their apparent dispensability in Vero cells may reflect the lack of an interferon response, as lipid droplets support antiviral immunity (66). Notably, other arenaviruses such as JUNV decrease lipid droplets and are sensitive to etomoxir (79), highlighting potential differences between Old World and New World arenaviruses.

Although FASN dependence has been reported for many viruses, this study is the first to demonstrate it in mammarenaviruses (80). Viral reliance on FASN varies widely, affecting multiple stages of infection, often through disrupted acylation of viral proteins. For example, in SARS-CoV-2 and MERS-CoV, reduced palmitoylation of structural proteins impairs infectivity and budding, while in CHIKV it affects replication complexes (81, 82, 83, 84, 85). HIV, which also produces a myristoylated matrix protein, can be rescued from FASN inhibition associated decreases in matrix myristoylation by C14:0 supplementation (86, 87). However, unlike HIV, neither C14:0 nor C16:0 restored r3LCMV or r3LCMV-LASV infection under FASN inhibition, indicating a distinct mechanism.

The mechanism by which C18:1 restores r3LCMV-LASV and r3LCMV infection during FASN inhibition remains unclear, but several lines of evidence argue against rescue via regeneration of Z myristoylation through β-oxidation of C18:1. FASN inhibition blocks β-oxidation, and C18:1 is a poor substrate for CPTI (88, 89), . Although oleic acid can stimulate β-oxidation (90), supplementation with C14:0, capable of restoring myristate in other systems (87), did not rescue infection. Additionally, β-oxidation of C18:1 would yield C14:1 (n-9), a rare fatty acid not typically used for myristoylation (91, 92, 93, 94, 95). Finally, while TVB-2640 inhibits infectious particle production for both r3LCMV and r3LASV, only LCMV Z shows altered localization consistent with loss of myristoylation, further arguing against this mechanism.

Instead, we suggest that a complex lipid species downstream of FASN synthesis is being depleted or acylation of a cellular protein is being inhibited, and that the C18:1 that rescues viral budding is being diverted into phospholipids or protein acylation pathways, even as a significant fraction also accumulates in lipid droplets. While FASN inhibition reduces lipid droplets, potentially causing ER stress that could contribute to viral inhibition (75), this is unlikely to be the primary mechanism, as inhibition of DGAT1 with A922500 also depletes lipid droplets but did not inhibit r3LCMV-LASV.

It is not without precedent that acylated cellular proteins might be crucial for virus infection (96, 97, 98). Esterification of oleic acid to proteins, or oleoylation, is not as well studied as palmitoylation or myristoylation, though many cellular proteins, such as H-Ras and GPCR are oleoylated (99, 100). However, inhibition of CPTI by etomoxir, which should highly decrease whole cell protein acylation (59), had no effect upon r3LCMV-LASV (Fig. 7C). Thus, we favor a model where FASN alters membrane composition through depletion of complex membrane lipids; budding of the arenaviral Z-protein could be affected by decreased saturation and membrane fluidity secondary to FASN inhibition, as many membrane proteins are known to prefer specific lipid species over others (101, 102). In the future, we can discern the destiny of supplemented C18:1 within the FASN-inhibited cellular system with stable ^13^C isotope fatty acids. To complement this, replication of mammarenaviruses in lipoprotein-depleted serum media with supplementation of multiple species of lipids could allow for disambiguation of the necessary lipids.

The primary experiments were conducted in Vero cells, which are commonly used for viral studies due to their lack of key antiviral factors, though this necessitates careful interpretation of results (103). Kidney cells are relevant to arenavirus infection, as high viral titers (104, 105) and associated pathology have been observed in kidney tissues (106, 107, 108, 109, 110). Because the liver is the main site of replication for Lassa virus, we also used hepatocellular carcinoma cell lines (Huh7 and Hepa1-6) with intact interferon responses (111, 112, 113), demonstrating that mammarenaviral sensitivity to FASN inhibitors is independent of IFN. However, cancer-derived cell lines may overestimate cytotoxicity, as they are particularly sensitive to FASN inhibition (114, 115, 116, 117, 118, 119, 120, 121).

In conclusion, we have identified that endogenous fatty acid synthesis is a cellular pathway that is critical for successful infection by arenaviruses. This insight expands basic virological knowledge and could inform the development of host-directed antiviral therapies. While not illuminated in our study, future research could identify which complex lipid species arenaviruses rely on for binding of Z to the plasma membrane, whether FASN-IFN regulation plays a role in the innate immune response of arenavirus infection, as well as whether FASN inhibitors restrict arenaviral infection in animal models.

## Data availability

Mass spectrometry data is available at MassIVE, under Accession MSV000100137.

## Supplemental data

This article contains supplemental data.

## Conflict of interest

The authors declare that they do not have any conflicts of interest with the content of this article.

## Supplemental Data

Supplemental Table 1 Table of lipid abundances identified through PCA analysis of HILIC-IM-MS data of Mock and LCMV-LASV infected VeroS cells. Statistics: -log_10_(q value), Two-stage step-up (Benjamini, Krieger, and Yekutieli), FDR = 1%.
